# Transposable element derived DNaseI-hypersensitive sites in the human genome

**DOI:** 10.1186/1745-6150-1-20

**Published:** 2006-07-20

**Authors:** Leonardo Mariño-Ramírez, I King Jordan

**Affiliations:** 1 National Center for Biotechnology Information, National Institutes of Health, Bethesda, MD 20894, USA

## Abstract

**Background:**

Transposable elements (TEs) are abundant genomic sequences that have been found to contribute to genome evolution in unexpected ways. Here, we characterize the evolutionary and functional characteristics of TE-derived human genome regulatory sequences uncovered by the high throughput mapping of DNaseI-hypersensitive (HS) sites.

**Results**

Human genome TEs were found to contribute substantially to HS regulatory sequences characterized in CD4+ T cells: 23% of HS sites contain TE-derived sequences. While HS sites are far more evolutionarily conserved than non HS sites in the human genome, consistent with their functional importance, TE-derived HS sites are highly divergent. Nevertheless, TE-derived HS sites were shown to be functionally relevant in terms of driving gene expression in CD4+ T cells. Genes involved in immune response are statistically over-represented among genes with TE-derived HS sites. A number of genes with both TE-derived HS sites and immune tissue related expression patterns were found to encode proteins involved in immune response such as T cell specific receptor antigens and secreted cytokines as well as proteins with clinical relevance to HIV and cancer. Genes with TE-derived HS sites have higher average levels of sequence and expression divergence between human and mouse orthologs compared to genes with non TE-derived HS sites.

**Conclusion:**

The results reported here support the notion that TEs provide a specific genome-wide mechanism for generating functionally relevant gene regulatory divergence between evolutionary lineages.

**Reviewers:**

This article was reviewed by Wolfgang J. Miller (nominated by Jerzy Jurka), Itai Yanai and Mikhail S.Gelfand.

## Open peer review

Reviewed by Wolfgang J. Miller (nominated by Jerzy Jurka), Itai Yanai and Mikhail S.Gelfand. For the full reviews, please go to the Reviewers' comments section.

## Background

Transposable elements (TEs) are DNA sequences capable of moving among chromosomal locations within the genome. TEs are copious genomic entities; at least half of the human genome sequence is derived from TE insertions [1,2]. While TEs were once thought to be purely selfish parasites concerned only with their own proliferation [3], there are now numerous examples of TE sequences that have been domesticated [4] to play some role for the host genomes in which they reside [5]. One way that TEs can achieve such a mutualistic status is through the donation of regulatory sequences that help control the expression of nearby host genes. For instance, recent genome-wide studies have shown that TEs can be found in gene-specific regulatory regions such as proximal promoters and untranslated regions as well as regulatory sequences that exert more global effects like scaffold/matrix attachment regions and locus control regions [6,7]. LINE elements, in particular, have been demonstrated to have genome-wide effects in lowering expression when inserted within transcribed regions [8]. Comparative sequence analyses have shown that many sequences from two particular human TE families have evolved under purifying selection, strongly suggesting a functional role related to gene regulation [9]. More specifically, numerous experimentally characterized cis-regulatory binding sequences have been shown to be derived from TE insertions [6,10], and a number of anecdotal cases of gene regulatory phenotypes governed by TE sequences have been confirmed [11,12]. At the same time, TEs are known to be among the least evolutionarily conserved elements in the human genome [1]. Indeed, TE activity and insertions often lead to the most substantial evolutionary differences between mammalian genome sequences [13,14]. Taken together with their ability to donate regulatory sequences, the lineage-specific nature of TEs suggests that they may provide a specific mechanism for driving the regulatory divergence between evolutionary lineages [10].

There are numerous experimental and computational efforts underway aimed at characterizing the non-coding portion of mammalian genomes [15]. Much of this work is focused on elucidating the location and nature of regulatory sequences that control the expression of nearby genes. An example of this kind of work is the large scale attempt, spearheaded by the National Human Genome Research Institute (NHGRI), to characterize a complete set of human genome DNaseI-hypersensitive (HS) sites . HS sites are associated with gene regulatory regions, for upregulated genes in particular, and mapping of HS sites is considered to be among the most reliable experimental methods for identifying regulatory sequences. HS sites have been shown to be associated with a variety of regulatory regions such as promoters, enhancers, suppressors, insulators, and locus control regions [16].

Using recently developed high throughput experimental methods, thousands of HS sites were cloned from human CD4+ T cells [17,18] and sequenced using massively parallel signature sequencing [19]; results were confirmed with real time PCR [20]. CD4+ T cells are a class of lymphocytes known as helper or effector T cells that serve to activate and direct other immune cells. CD4+ T cells are one of the primary targets of HIV infection, and depletion of these cells leads to AIDS. Thus, the HS sites mapped to the human genome by the NHGRI should correspond to sequences that regulate gene expression related to CD4+ T cell mediated immune response.

In this report, we have taken advantage of the genome-wide mapping of HS sites in order to evaluate the contribution of TEs to human gene regulatory sequences. The extent of TE-derived HS sites in the human genome was characterized, and the evolutionary conservation levels of TE-derived versus non TE-derived HS sites were compared. In addition, the expression and functional characteristics of genes with TE-derived HS sites were evaluated along with the evolutionary divergence of their sequences and expression patterns. The results reported here indicate that TEs have provided numerous functionally relevant HS sites to the human genome, and these regulatory sequences have played a role in driving functional divergence along the human evolutionary lineage.

## Results and discussion

### Human genome DNaseI-hypersensitive sites

A total of 14,216 DNaseI-hypersensitive (HS) sites, covering ~4.2 megabases of DNA, are mapped to the hg17 version (NCBI build 35) of the human genome sequence. These sites consist of clusters of two or more experimentally characterized HS sites that map within 500 bp of each other. These HS sites were defined in CD4+ T cells and are presumed to be functionally relevant with respect to the regulation of gene expression in these cells. Given the functional role played by HS sites, they are expected to be anomalously conserved in terms of their levels of sequence divergence. This is because the evolution of functionally important sequences is constrained by purifying selection (*i.e*. the removal of deleterious variants). Indeed, this idea is the basis of the phylogenetic footprinting approach that identifies putatively functional genomic elements by virtue of their sequence conservation [21,22]. The expectation that HS sites should be evolutionarily conserved was tested using the binary characterization of human genome positions as conserved or non-conserved based on analysis with the program phastCons [23]. PhastCons employs a probabilistic hidden Markov model (HMM) that represents the levels of DNA substitution at each site in the genome and how these levels change among sites. The phastCons results used here were based on a human query anchored multiple sequence alignment (MSA) of 17 vertebrate genomes. This MSA was assembled with the program multiz [24] from whole genome pairwise alignments generated using blastz [25]. The HMM used by phastCons employs a single phylogenetic tree for all sites with the branch lengths free to vary across sites. The HMM has two states – conserved and non-conserved – based on the values of the branch length scaling parameter estimated from the data. Alignment sites (segments) are predicted as being conserved if they are significantly more likely to have been generated by the conserved state of the HMM.

**Figure 1:**
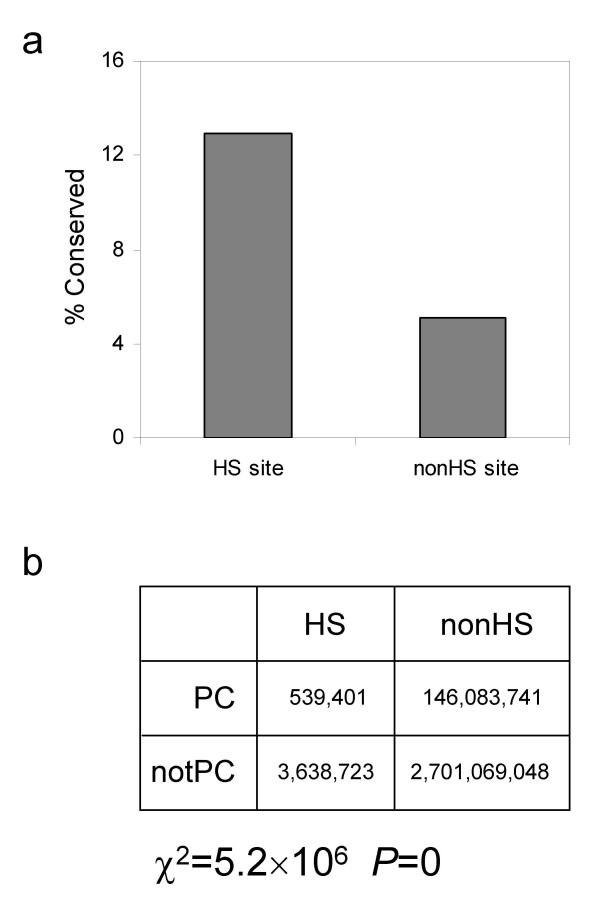
**DNaseI-hypersensitive site conservation**. a) The percent of phastCons (PC) denoted conserved positions for DNaseI-hypersensitive (HS) versus non DNaseI-hypersensitive (nonHS) sites in the human genome. b) 2 × 2 contingency table for evaluating the difference in evolutionary conservation between HS and nonHS sites.

All HS and non HS sites in hg17 were evaluated with respect to their phastCons conservation designation. HS sites in the human genome were found to be far more conserved, on average, than sites non HS sites (Figure 1a): ~13% of HS sites are conserved compared to ~5% of non HS sites. This comparison is conservative because the non HS sites include numerous exons, most of which are conserved. The figure of 5% conserved non HS sites is consistent with previous estimates for the proportion of the human genome evolving under purifying selection [23]. The greater than two-fold difference in conservation between HS and non HS sites, together with the large number of genome positions evaluated, yields a highly statistically significant difference (χ^2 ^= 5.2 × 10^6^, *P *= 0; Figure 1b). These results are consistent with previous reports that HS sites are i-enriched in human genome regions that align with mouse genomic sequence [17] and ii-often located near sequences conserved in multiple species [18]. However, it should be noted that the majority of HS sites (~87%) analyzed here are not considered conserved by the criteria employed by phastCons. Thus, many of these sites, which are likely to be functionally important, might not be detected using a naïve phylogenetic footprinting approach.

### Transposable element derived hypersensitive sites

**Figure 2:**
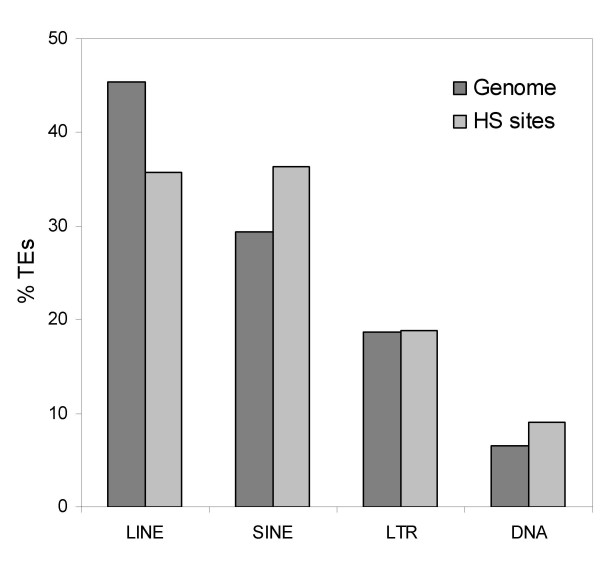
**Relative percentages of four transposable element (TE) classes**. Percentages of the four classes are shown for the human genome as a whole (dark grey) and for HS sites only (light grey).

The contribution of TEs to HS sites in the human genome was evaluated by comparing the results of a RepeatMasker [26] analysis of hg17 to the mapped locations of the NHGRI HS sites. There are numerous TE-derived HS sites in the human genome: 3,229 HS sites (~23%) include TE sequence and 11% of all positions covered by HS sites (454,564 bp) are derived from TEs. This substantial fraction of HS sites derived from TEs is likely to be an underestimate since some TEs have diverged beyond recognition and others may not be covered by the RepBase library [27] employed by RepeatMasker. The relative frequency distribution of TE classes found to donate HS sites is largely similar to genomic distribution of TEs with a slight over-representation of SINEs and according under-representation of LINEs (Figure 2). This probably reflects the fact that SINEs, such as Alus, are over-represented in GC and gene rich regions of the genome, while LINEs tend to be found in relatively gene poor AT-rich regions.

**Figure 3:**
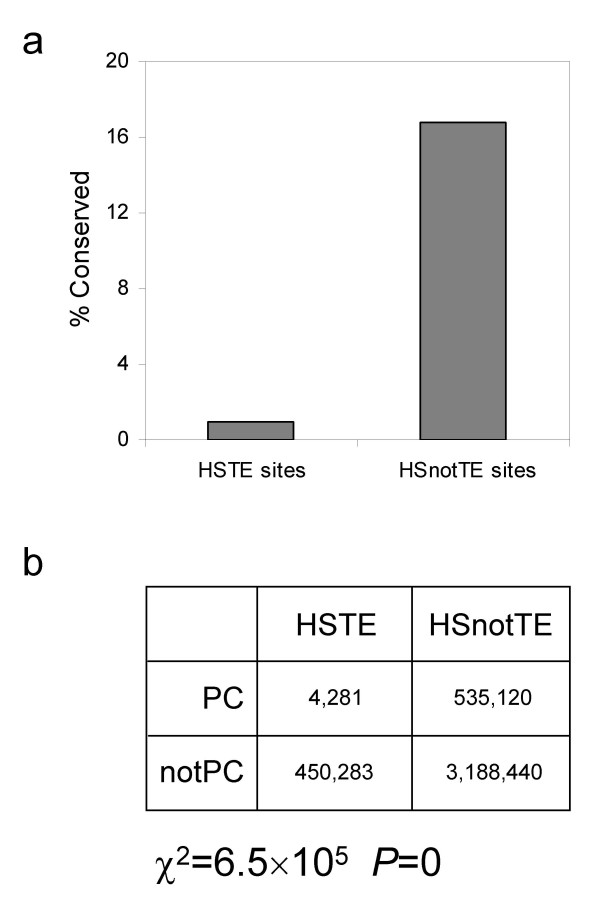
**Evolutionary conservation of TE-derived versus non TE-derived HS sites**. a) The percent of phastCons (PC) denoted conserved positions for TE-derived (HSTE) and non TE-derived (HSnotTE) sites in the human genome. b) 2 × 2 contingency table for evaluating the difference in evolutionary conservation between HSTE and HSnotTE sites.

The evolutionary conservation of TE-derived HS sites was compared to that of non TE-derived HS sites using the same phastCons-based approach described previously. HS site positions derived from TEs are far less conserved (<1%) than non TE-derived HS site positions (~17%; Figure 3a), and the difference is highly statistically significant (χ^2 ^= 6.5 × 10^5^, *P *= 0; Figure 3b). This finding is consistent with the highly lineage-specific nature of TE-sequences. Even in the human genome, where they are relatively ancient, TEs are among the least conserved of all sequences [1]. The lack of evolutionary conservation for TE-derived HS sites can be taken to suggest the formal possibility these sites are not functionally relevant. However, the analysis of gene expression data described below indicates that TE-derived HS sites do in fact play a functional role related to CD4+ T cell-specific gene regulation. Thus, it may be the case that TE-derived HS sites provide a specific mechanism that leads to lineage-specific regulatory phenotypes, which in turn are important for the generation of higher level phenotypic differences between species. This interpretation is consistent with what has previously been shown for TE-derived cis-regulatory sequences with demonstrable roles in gene regulation, a majority of which also show little or no evidence of evolutionary conservation [10].

### Gene expression

**Figure 4:**
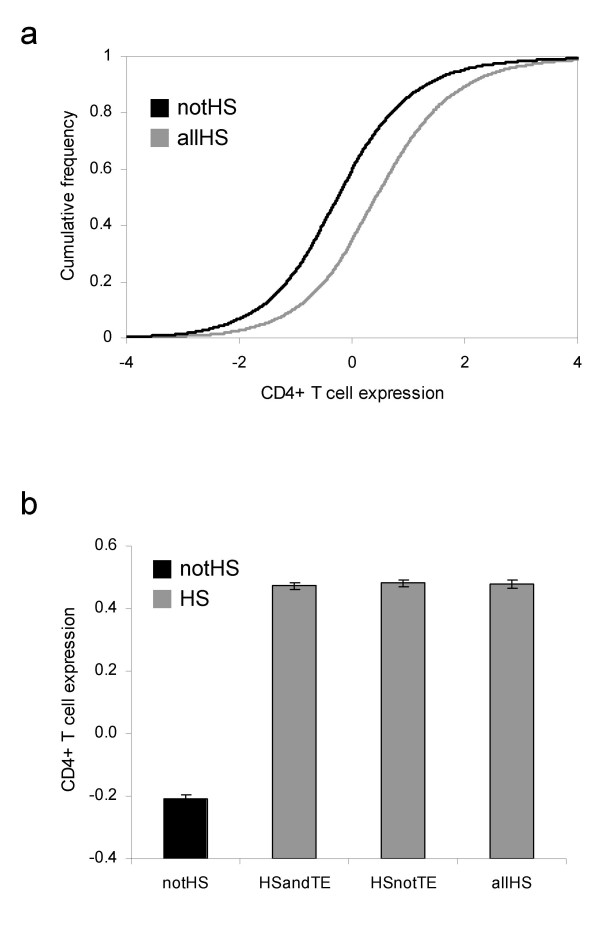
**Comparison of CD4+ T cell expression levels for different classes of human genes**. a) Cumulative frequency distributions of CD4+ T cell expression for genes do not have any HS sites (notHS) and genes with HS sites (allHS). b) Comparison of average CD4+ T cell expression levels for without (notHS) and with (allHS) HS sites. Genes with HS sites were further broken down into groups of genes with TE-derived (HSandTE) and not TE-derived (HSnotTE) HS sites.

The HS sites analyzed here were cloned from CD4+ T cells. As such, the HS site locations in the human genome should correspond to genes that are upregulated in CD4+ T cells. This prediction was tested using Affymetrix microarray gene expression data from the Novartis Research Foundation SymAtlas (GNF2) [28]. The locations of all hg17 mapped HS sites were considered with respect to the locations of the so-called known genes from the UCSC genome browser. HS sites were associated with a gene if they mapped within 1-kb of the start or end of transcription for that gene, and Affymetrix probe identifiers were associated with individual genes. The relative levels of CD4+ T cell-specific gene expression were compared for genes that have co-located HS sites and those that have no HS site. Genes associated with HS sites have significantly higher relative CD4+ T cell expression (μ = 0.48) than non HS site (μ = -0.21) genes (Student's t-test *P *= 4.5e-264; Mann-Whitney U test, *P *= 8.2e-300; Figure 4a and 4b). This is consistent with a functional role for these HS sites in driving gene expression in CD4+ T cells. Similar results, finding HS sites near genes with demonstrable CD4+ T cell expression, were reported previously [17,18]. In addition, there is no real difference in the levels of CD4+ T cell expression between TE-derived (μ = 0.48) and non TE derived (μ = 0.47) HS sites (Student's t-test, *P *= 0.82, Mann-Whitney U test, *P *= 0.33; Figure 4b). This finding underscores the functional relevance of TE-derived HS sites: they too appear to drive CD4+ T cell specific expression.

### Gene function

**Figure 5:**
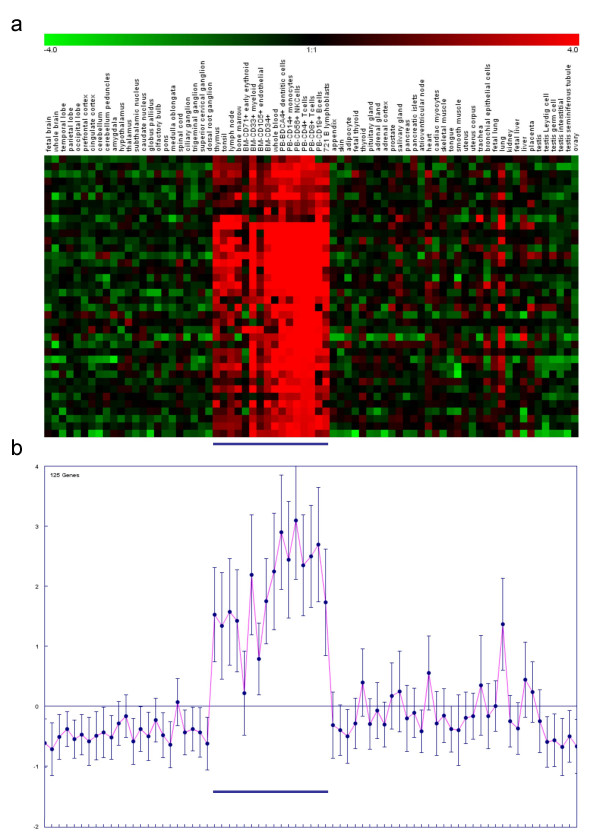
**Gene expression patterns for a cluster of coexpressed genes with TE-derived HS sites**. a) View of relative expression levels across conditions for a subset of cluster 2 genes, color coded according to over (red) and under (green) expression. b) Centroid view with cluster 2 expression averages.

The GNF2 expression data was also used to guide functional evaluation of genes with TE-derived HS sites. Condition-specific gene expression patterns, across 73 (non-cancerous) human tissue types and cell lines, were used to group this set of genes with k-means clustering [29]. A total of 25 clusters, containing genes with similar expression profiles, were resolved in this way, and five clusters (2, 9, 15, 19 & 25) containing genes with pronounced CD4+ T cell over-expression were chosen for functional analysis. Genes with relatively high expression in CD4+ T cells also tend to be over-expressed in other immune related tissues and cells such as blood, bone marrow and lymphoblasts (Figure 5). These coherent expression patterns strongly suggest a functional role related to immune response, and the annotation of the genes in question bear this interpretation out.

**Figure 6:**
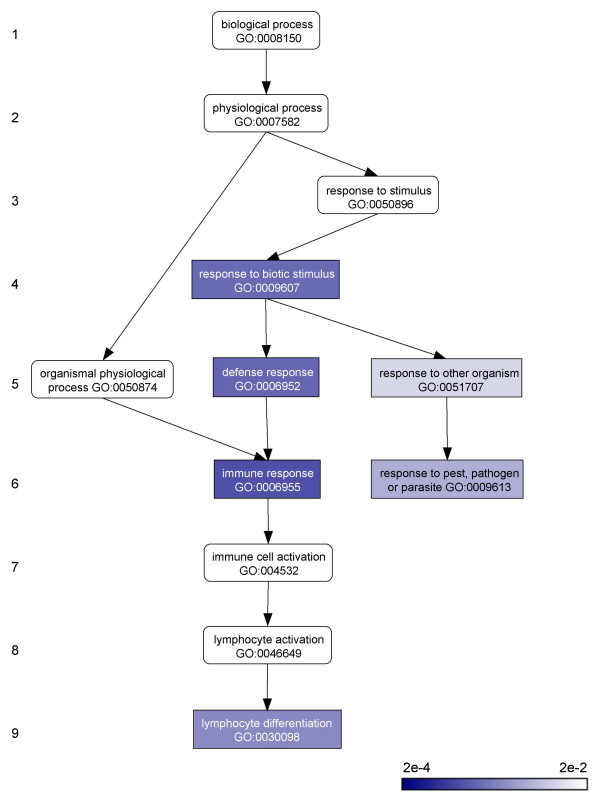
**Path on the GO biological process graph leading to overrepresented functions related to immune response**. Hierarchical levels of the directed acyclic GO graph are shown on the left. Nodes in the graph represent individual GO annotation terms. Nodes are color coded according to the statistical significance level for their overrepresentation. Edges in the graph represent parent-child relationships between GO terms.

Gene ontology (GO) annotation terms were mapped to individual genes, and the gene members of each cluster were evaluated for the over-representation of specific GO (biological process) terms. GO terms found to be significantly over-represented in two or more clusters are shown in Table 1. When the gene expression clusters are considered from this functional annotation perspective, they resolve into cluster- and function-specific groups. Most prominent among these is the immune response group, which unites clusters 2 and 25 (as well as 19 to a lesser extent) and contains six immune related GO terms. The hierarchical (parent-child) relationships among these immune related terms on the GO graph are shown in Figure 6. The most significantly over-represented GO term covers genes specifically involved in immune response (GO:0006955).

**Table 1:** Overrepresented GO terms from genes with TE-derived HS sites

***Clusters***^1^	***GO id***^2^	***GO level***^3^	***GO name***^4^	***Counts***^5^	**P*-values***^6^
Immune response group					
2	GO:0009607	4	response to biotic stimulus	21	4.9 × 10^-7^
25				8	1.7 × 10^-2^
2	GO:0006952	5	defense response	20	1.0 × 10^-6^
25				8	1.6 × 10^-2^
2	GO:0006955	6	immune response	20	3.6 × 10^-7^
25				8	1.3 × 10^-2^
2	GO:0051707	5	response to other organism	11	1.8 × 10^-4^
25				5	3.3 × 10^-2^
2	GO:0009613	6	response to pest, pathogen or parasite	11	1.0 × 10^-4^
25				5	2.7 × 10^-2^
2	GO:0030098	9	lymphocyte differentiation	3	6.2 × 10^-3^
19				3	1.6 × 10^-2^
Regulation group					
15	GO:0050789	2	regulation of biological process	40	4.4 × 10^-8^
25				17	7.5 × 10^-3^
15	GO:0050794	3	regulation of cellular process	39	2.2 × 10^-8^
25				17	4.1 × 10^-3^
15	GO:0050791	3	regulation of physiological process	38	2.2 × 10^-8^
25				15	1.3 × 10^-6^
15	GO:0051244	4	regulation of cellular physiological process	38	1.3 × 10^-8^
25				15	1.3 × 10^-2^
Metabolism group					
15	GO:0050875	3	cellular physiological process	73	6.5 × 10^-4^
19				88	3.8 × 10^-3^
15	GO:0044238	4	primary metabolism	63	1.7 × 10^-6^
19				67	4.8 × 10^-3^
15	GO:0044237	4	cellular metabolism	63	9.0 × 10^-6^
19				71	1.3 × 10^-3^
9	GO:0043170	4	macromolecule metabolism	26	2.8 × 10^-2^
15				37	2.0 × 10^-2^
19				50	9.1 × 10^-4^
9	GO:0043283	5	biopolymer metabolism	18	2.8 × 10^-2^
15				30	8.0 × 10^-5^
19				35	5.1 × 10^-4^
Cell death group					
2	GO:0043067	6	regulation of programmed cell death	7	7.8 × 10^-3^
19				7	3.7 × 10^-2^
2	GO:0042981	7	regulation of apoptosis	7	7.8 × 10^-3^
19				7	3.7 × 10^-2^

^1^Gene coexpression, based on k-means analysis, cluster identifier.

^2^Biological process Gene Ontology (GO) annotation term.

^3^Hierarchical level (descending from 1) in the biological process GO directed acyclic graph.

^4^Name assigned to the GO term

^5^Number of genes in each cluster associated with the GO term.

^6^*P*-value associated with the over-representation of the GO term in each cluster

The functions of the individual genes that have TE-derived HS sites as well as immune response related expression patterns and functional annotation (GO:0006955) are shown in Table 2. This example further emphasizes the specific functional relevance of TE-derived HS sites. For instance, there are a number of T cell receptor antigens, such as CD6, CD53, CD74 and CD97, with T cell-specific expression patterns influenced by TE insertions. These antigens are involved in processes like cell-cell adhesion and signalling cascades that govern the development and proliferation of immune cells. There are also cases of excreted cytokines (*e.g*. CKLF & CCLF) that are involved in the inflammatory response by serving as potent chemoattractants and stimulants for T cells. Finally, there are clinically relevant genes with TE-derived HS sites, such as CCL5, which suppresses HIV by serving as a natural ligand for the HIV coreceptor CCR5, and BCL2, which blocks the apoptotic death of lymphocytes and can lead to lymphoma when constitutively expressed.

**Table 2:** Immune response (GO:0006955) genes with TE-derived HS sites

***Cluster***^1^	***Name***^2^	***Accn***^3^	***Description***^4^	***HS site***^5^	***TE***^6^
2	CD97	NM_078481	leukocyte antigen; receptor involved in both cell adhesion and signalling processes early after leukocyte activation	12460_5	SINE/MIR/MIR
2	IGLC2	BC073786	Ig light-chain, partial Ke-Oz- polypeptide, C-term; immunoglobulin lambda constant region 2	13851_3	LTR/ERVL/LTR16A
2	IGLC1	BC070353	constant region of lambda light chains	13851_3	LTR/ERVL/LTR16A
2	MR1	CR602405	major histocompatibility complex, class I-related	1311_2	LINE/L2/L2
2	ADORA2A	NM_000675	adenosine receptor subtype A2a; G-protein coupled receptor; reduces the activation status of inflammatory cells	13875_2	SINE/MIR/MIR3
2	CKLF	NM_016951	chemokine-like factor (cytokine); essential role in the immune and inflammatory responses; potent chemoattractant for neutrophils, monocytes and lymphocytes	10591_2	SINE/Alu/AluSg
2	KLF6	NM_001008490	Kruppel-like factor 6; core promoter guanine-rich element binding protein; transcriptional activator	6794_2	SINE/MIR/MIRb
2	IL16	CR749286	interleukin 16; lymphocyte chemoattractant factor; cytokine; modulator of T cell activation; mediated by CD4	10023_2	DNA/MER1/MER117
2	ST6GAL1	NM_173216	sialyltransferase 1 (beta-galactoside alpha-2,6-sialytransferase); role in T-cell death; generation of cell-surface carbohydrate determinants and differentiation antigens	3308_3	SINE/MIR/MIR3
2	HLA-E	NM_005516	HLA class I histocompatibility lymphocyte antigen, E alpha chain; immunoregulatory role for cytotoxic T-lymphocytes	4584_2	DNA/MER1/Charlie1
2	IL21R	NM_181078	interleukin 21 receptor; type I cytokine receptor; transduces the growth promoting signal of IL21, and is important for the proliferation and differentiation of T cells, B cells, and natural killer (NK) cells	10408_3	SINE/MIR/MIRb
2	ITGB2	NM_000211	leukocyte cell surface adhesion glycoprotein; complement receptor C3 beta-subunit; integrin beta 2; macrophage antigen 1; facilitates inflammatory cell recruitment	13756_2	LINE/L1/LIME4a
2	FYB	NM_001465	FYN-binding protein; adhesion and degranulation-promoting adaptor protein; adaptor helps form immunological synapse between T cell and antigen-presenting cell; mediates signaling from the T cell antigen receptor to integrins	3896_2	SINE/MIR/MIR3
2	IGHG1	BC024289	immunoglobulin heavy constant region gamma 1; involved in antigen binding and immune response	9667_2	LINE/L1/L1
2	PTPRC	Y00062	protein tyrosine phosphatase receptor; leukocyte-common CD45 antigen; essential regulator of T- and B-cell antigen receptor signaling; regulator of cytokine receptor signaling; involved in hematopoiesis	1347_2	LINE/L1/L1ME
2	LCP2	NM_005565	lymphocyte cytosolic protein 2; adaptor or scaffold protein that promotes T cell development and activation as well as mast cell and platelet function.	4315_2	SINE/Alu/AluSx
2	CD53	NM_000560	glycoprotein leukocyte cell surface antigen; contributes to the transduction of CD2-generated signals in T cells and natural killer cells; role in T cell growth regulation	964_2	LINE/L2/L2
2	DOCK2	NM_004946	dedicator of cytokinesis 2; hematopoietic cell-specific CDM family protein essential for lymphocyte chemotaxis; mediates T cell receptor-induced activation of Rac2 and IL-2	4313_2	SINE/Alu/AluSx SINE/MIR/MIR
2	CD74	BC024272	cell surface antigen; major histocompatibility complex, class II invariant chain; involved in NF-kappaB activation and interleukin-8 production	4254_2	DNA/MER1/Charlie8
2	MX1	NM_002462	myxovirus resistance protein 1; interferon inducible; role in host defense against viruses	13686_3	DNA/MER1/MER5A
25	CD6	U66145	glycoprotein cell surface antigen; involved in lymphocyte activation and thymocyte development; role in maturation of the immunological synapse		DNA/MER1/MER113
25	PLA2G4B	NM_005090	phospholipase enzyme secreted by neutrophils; produces arachidonic acid used for the biosynthesis of leukotriene in inflammatory response	9758_2	SINE/MIR/MIRb SINE/Alu/AluY
25	BCL2	NM_000633	B-cell lymphoma protein 2; blocks the apoptotic death of some cells such as lymphocytes	12021_2	LINE/L1/HAL1
25	TRA@	BC063432	T cell antigen receptor alpha locus; involved in thymocyte developement	9169_2 9171_2 9174_4	LINE/L1/L1ME2 SINE/MIR/MIR LINE/L2/L2
25	CCR9	NM_031200	chemokine (C-C motif) G protein coupled receptor; regulator of thymocytes migration and maturation in normal and inflammation conditions; functional specialization of immune responses in different segments of the gastrointestinal tract	2827_2	LINE/L1/L1ME4a
25	TCF7	NM_003202	T cell specific transcription factor; regulates T cell development and peripheral T cell differentiation	4139_4	LINE/CR1/L3
25	CCL5	NM_002985	T cell specific chemokine (C-C motif) ligand; chemoattractant for blood monocytes, memory T helper cells and eosinophils; causes release of histamine from basophils and activates eosinophils;	11206_2	LINE/L1/L1MB3
25	LAT	NM_001014987	linker for activation of T cells; phosphorylated following activation of the T-cell antigen receptor signal transduction pathway; acts as a docking site and recruits multiple adaptor proteins and downstream signaling molecules into multimolecular signaling complexes	10425_2	SINE/Alu/AluJb SINE/MIR/MIR

^1^Gene coexpression cluster from which the gene is taken.

^2^Official gene name symbol from the HUGO Gene nomenclature committee

^3^Representative mRNA Genbank accession taken from USC genome browser knownGene table.

^4^Brief description of the gene function

^5^NHGRI DNase-I hypersensitive site identifier taken from the UCSC genome browser nhgriDnaseHs table.

^6^Class/family/name of the transposable element (TE) sequence colocalized with the HS site

### Comparative genomics

Gene expression and function analysis point to the importance of genes with TE-derived HS sites. However, these same TE-derived HS sites are not evolutionarily conserved. This suggests that TE-derived HS sites may be important in generating functional differences between evolutionary lineages. Comparative analyses of gene sequence and expression divergence between human and mouse orthologs were performed to evaluate this possibility. Genes with HS sites were divided into those with TE-derived sites and those with non TE-derived sites. These two gene sets were mapped to 9,105 pairs of human-mouse orthologous gene pairs described previously [30], each member of which has GNF2 expression data. Proteins encoded by genes with TE-derived HS sites have slightly higher levels of sequence divergence (0.147 substitutions per site) compared to those encoded by genes with non TE-derived HS sites (0.138 substitutions per site). The difference between these average substitution rates is only marginally significant (Student's t-test, *P *= 0.12, Mann-Whitney U test, *P *= 0.08). Genes with TE-derived HS sites also have slightly greater evolutionary differences in CD4+ T cell expression (1.005) than those with non TE-derived HS sites (0.948) as measured by comparison between human and mouse orthologs. The difference in CD4+ T cell expression for TE-derived versus non TE-derived HS site genes is only marginally significant as well (Student's t-test, *P *= 0.08, Mann-Whitney U test, *P *= 0.11). However, taken together, the differences in evolutionary divergence at the sequence and expression level are consistent with the idea that TE-derived HS sites help to drive evolutionary changes between lineages. The magnitude of this effect is fairly small though, just under 10% difference for both sequence and expression, contributing to the marginal significance in each case and indicating that many other factors are in play with respect to the evolutionary divergence of these genes and phenotypes.

## Conclusion

TEs contribute numerous HS sites to the human genome. While TE-derived HS sites are not evolutionarily conserved, they are functionally relevant, as demonstrated by analyses of gene expression and functional annotations. This distinction between conservation and function can be taken to suggest that TEs provide a specific mechanism for driving regulatory differences between evolutionary lineages, and comparative genomics data bear this notion out to some extent. Genes with TE-derived HS sites are slightly more divergent than those non TE-derived HS sites in terms of both sequence and CD4+ T cell expression. The results reported here point to genome-scale effects that TEs have had in shaping the regulatory evolution of their host genomes.

## Methods

### Human genome sequence

The May 2004 release – National Center for Biotechnology Information (NCBI) build 35 – of the human genome reference sequence [1] was analyzed using the UCSC genome browser [31]. The UCSC database containing this particular release and all associated data is referred to as hg17. The chromosome coordinates of various attributes mapped onto the hg17 genome sequence were downloaded using the UCSC Table Browser retrieval tool [32]. The Table Browser retrieval tool was also used to perform a number of logical set operations between specific tables (see below) that allowed for the identification of co-located genomic attributes.

### Hypersensitive sites

DNaseI-hypersensitive sites (HS) from CD4+ T cells were characterized as described [17-19]. The HS sites have been mapped onto the hg17 sequence and their genome coordinates were retrieved from the table named *nhgriDnaseHs*. Only clusters of more than on HS site that map within 500 bp of each other are mapped onto hg17.

### Transposable elements

The locations and identities of all hg17 TE sequences were characterized using the RepeatMasker program [26], which uses the RepBase library [27] of repeat sequences. The genome coordinates, along with the class, family and name designations, for TEs were retrieved from the table named *rmsk*.

### Gene expression and function

Human and mouse microarray gene expression data are from the Genomics Institute of the Novartis Research Foundation SymAtlas (GNF2) [28]. These data were retrieved from the table named *gnfAtlas2*. This table stores relative expression values for 79 different human tissues and/or cell types (conditions). Relative expression levels are computed as follows: Two replicate microarray experiments were performed for each condition, and for each individual probe, the expression signal intensity values were averaged for each of the 79 pairs of experiments. Then, for each probe, each of the 79 condition-specific averages was normalized by the median of all values for that probe to determine relative expression levels; these ratios were log2 normalized prior to analysis. Affymetrix probe identifiers are mapped to UCSC Genome Browser known genes and these data were retrieved from the table named *knownToGnfAtlas2*. The known genes are based mRNA data from the NCBI Reference Sequence (RefSeq) database and GenBank along with protein data from UniProt. The chromosome coordinates for the known genes were retrieved from the table named *knownGene*. After probe-to-gene mapping, relative CD4+ T cell expression levels were compared for different sets of human genes. Gene expression profiles were visualized and clustered, by k-means clustering, using the program Genesis [29]. The Pearson correlation coefficient was used to compute pairwise similarities between gene expression profiles. Individual clusters with high relative levels of CD4+ T cell expression, as well as high expression in related tissue-types, were chosen for further functional analysis. Clusters (*i.e*. groups of genes) were evaluated with respect to overrepresented Gene Ontology (GO) [33] functional annotation terms using the program GOstat [34]. The biological process subset of the GO hierarchy was used along with the European Bioinformatics Institute (EBI) human GO mapping. A 2 × 2 contingency table was used to compare the relative frequency of GO terms in the coexpression cluster test set (observed) versus the relative frequency of GO terms in the background set of all human GO terms (expected) using the χ^2 ^test, or the Fisher's exact test is the expected value<5. The Benjamini False Discovery Rate correction for multiple testing was used to adjust the resulting *P*-values. GO annotation terms that were found to be overrepresented in two or more different clusters were chosen for further analysis. Individual gene functions were explored using the NCBI Entrez Gene database . The graphical (parent-child) relationships among GO terms related to immune response were characterized using the GeneInfoViz program [35]. GO term significance levels were color coded using the program matrix2png [36].

### Comparative genomics

Conserved hg17 genomic sites were characterized using the phastCons program [23]. Alignment, using blastz [25] and multiz [24], and comparison among 17 vertebrate genome sequences were used to characterize conserved sites. The genome coordinates for conserved sites were retrieved from the table named *phastConsElements17way*. Absolute differences between the relative levels of CD4+ T cell expression were calculated for human and mouse orthologous genes pairs described previously [30]. The evolutionary divergence between human and mouse orthologous proteins was measured as the number of substitutions per site using the Poisson Correction distance [37].

## Reviewers' comments

### Reviewer's report 1

Wolfgang J. Miller, Laboratories of Genome Dynamics, Center of Anatomy and Cell Biology, Medical University of Vienna, Austria (nominated by Jerzy Jurka, Genetic Information Research Institute, Sunnyvale, CA, USA)

Reviewer comments:

This paper provides compelling evidence that TEs did contribute significantly to the evolution of novel regulatory sections in humans and other organisms by using an elegant and innovative combination between genomics with microarray gene expression data analyses. The authors show that up to one-fourth of the human DNAse I hypersensitive sites actually stem from TEs that serve important immune response functions in especially rapidly evolving genes. Due to the extreme lineage-specific expansion/silencing dynamics of TEs in different host systems absence of evolutionary conservation of TE-derived HS sites as reported here is not surprising. Therefore these data are not contradicting their functional relevance as important *cis*-regulatory sections but demonstrate that mobile DNAs in general do provide a highly attractive repertoire of structural and functional information patterns to the host. Even after their successful inactivation via host-directed silencing mechanisms such TE-derived cis-regulatory sections can if proven successful be adopted by the host genome for serving novel and innovative regulatory functions. It would be highly interesting to perform comparative genomic analyses between human and chimpanzee orthologous TE-derived HS sites in the near future.

#### Author's response

*We would like to thank Dr. Miller for taking the time and effort to review our manuscript. Dr. Miller suggests comparative genomic analyses between human and chimpanzee orthologous TE-derived HS sites. This is a good idea and may help to settle an issue, raised by Dr. Itai Yanai (Reviewer #2 below), concerning the role of TE-sequences as space holders versus the actual contribution of TE sequences to human gene regulation*.

### Reviewer's report 2

Itai Yanai, Department of Molecular and Cellular Biology, Harvard University, Cambridge, MA, USA

Reviewer Comments:

Marino-Ramirez and Jordan show in this paper that HS sites are significantly more conserved in sequence than non-HS sites although HS sites containing TE-derived genes are far less conserved that HS sites lacking TE's. In terms of gene expression, the authors show that TE-derived genes and non-TE derived genes in HS sites are as likely to be expressed in CD4+ T cells. Taken together, these results lead to the conclusion that TE's are useful in promoting gene expression evolution. This is an interesting notion. I imagine that TE's insertion may be instrumental in modulating expression patterns by altering the spacing among transcription binding sites as well as disrupting some motifs through their insertion. Thus their effects on gene expression may be conferred solely by their role as space holders consequently freeing the actual TE sequence to drift.

Indeed the authors also find that TE-derived genes evolve slightly faster in terms of sequence and expression. However the signal is so weak that it places into question the generality of this finding. One straightforward interpretation is that since in all likelihood most TE's that happen to lie at HS sites do not contribute to the evolution of gene expression, these dilute the signal to its observed weak level.

Overall, these findings are important and should further prompt research to attempt to distinguish those TE's which contribute to genomic function from those that do not.

#### Author's response

*We would like to thank Dr. Yanai for taking the time and effort to review our manuscript. Dr. Yanai proposes the interpretation that "most TE's that happen to lie at HS sites [that] do not contribute to the evolution of gene expression." Indeed, the evolutionary divergence in CD4+ T cell expression levels for genes containing TE-derived HS sites is only marginally greater than that seen for genes with non TE-derived HS sites, and we point this out in the text of the manuscript. However, the functional relevance of TE-derived HS sites is strongly supported by their association with genes that have relatively (significantly) higher levels of CD4+ T cell expression *(Figure 4).*In addition, genes with TE-derived HS sites have slightly higher sequence substitution rates, on average, than genes with non TE-derived HS sites. Taken together, these lines of evidence point to a potential role for TE-derived HS regulatory sites in facilitating the evolutionary divergence of human genes. To more carefully examine this issue, we plan to investigate regulatory changes of genes with TE-derived HS sites across species at different levels of evolutionary divergence from the human lineage*.

*Dr. Yanai also raises an interesting point about the role that TE sequences may play as 'space holders' as opposed to contributing specific regulatory sequences. Spatial changes among promoter elements caused by TE insertions may certainly have important functional consequences. On the other hand, there are a number of known, experimentally verified, cases of TE sequences providing specific cis-regulatory binding sequences. We are currently investigating the relative rates of evolution for TE sequences that co-locate with regulatory regions versus those that do not, among species that cover a range of evolutionary divergence from human, to further investigate this possibility*.

### Reviewer's report 3

Mikhail S.Gelfand, Department of Bioinformatics, Institute of Information Transfer Problems, Russian Academy of Science, Moscow, Russia

Reviewer Comments:

The paper reports analysis of hypersensitive sites (HSs), comparing HSs containing transposable elements (TEs) and HSs in general. Given that HSs are likely to correspond to regulatory regions and tend to be conserved, the large fraction of TE-containing HSs is surprising. Still, even the latter are shown to be functionally relevant. This leads to an important conclusion about the role of TEs in the evolution of regulation.

A main problem of this study is the deficit of controls. Basically, only two samples are analyzed, TE-HS and nonTE-HS. For instance: 23% HSs contain TEs and 11% HS positions are covered by TEs (page 7) – is it a lot or not? What would be expected if there were no correlation? (given that >50% of the human genome is TE-derived, I suspect that, not surprisingly, HS tend to avoid HS – but what about other classes of functional regions?)

There is no difference in gene expression between TE-HS and nonTE-HS sites (page 9) – this is nice, but, again, what about other classes of genes, e.g. dependent on the degree of conservation in their upstream regions.

Section "Gene function": what genes were subject to clustering in order to find over-represented GO annotations – all HS genes? TE-HS and nonTE-HS genes separately? Further, in the last paragraph of this section, it should be explicitly mentioned that it describes just an example, not an exhaustive list.

Section "Comparative genomics": missing controls are genes without HS in the same GO categories and just genes without HS: without it, it is difficult to appreciate the difference between the TE-HS and nonTE-HS genes. On the other hand, the results of this paragraph are quite interesting: assuming that TE-HS genes recently experienced a change in regulation (resulting in change in expression), one could expect positive selection towards a new role. It might be interesting to apply the McDonald-Kreitman test to check this hypothesis. But again, more controls with other classes of genes are needed.

Overall, I think that, although the obtained results are interesting, they are somewhat preliminary. To make the emerging picture much less shallow and enhance the authors' main point about the contribution of TEs to the evolution of regulation in the human genome, they should consider, where appropriate, the following control groups of genes: genes without HS, genes with non-conserved (but not TE-derived) HS, non-HS genes in the same GO categories as identified in the "Gene function" section.

#### Author's response

*We would like to thank Dr. Gelfand for taking the time and effort to review our manuscript. Dr. Gelfand calls attention to a 'deficit of controls', in several places, pointing out that only two samples are analyzed: TE-HS versus non TE-HS containing genes. Importantly, we have analyzed a third class of genes, namely those that do not have any co-located HS sites characterized in CD4+ T cells (non HS). This latter class of genes has significantly lower levels of CD4+ T cell expression than either class of HS site containing genes (TE-derived or non TE-derived*; Figure 4).*This underscores the functional relevance of both classes of HS sites characterized in CD4+ T cells, TE-derived and non TE-derived, with respect to CD4+ T cell specific expression. Additional analysis of this third class of genes yields slightly more ambiguous results related to the evolutionary divergence of human gene expression patterns. Non HS containing genes have average levels of CD4+ T cell expression divergence that are intermediate to those seen for TE-HS and non TE-HS containing genes. This is in keeping with the relatively weak signal seen for the differences in the average evolutionary divergence of CD4+ T cell expression levels (as well as gene sequence divergence) across different classes of genes, and related issues were raised by the other reviewers. We have been careful to point out this caveat in the manuscript and plan to perform additional evolutionary comparisons (as described in the answers to Reviewer 1 & 2 above), for instance on more closely related species, which may help to resolve the issue of the contribution of TE-derived HS sites to host gene regulatory divergence*.

*The point raised about 11% of HS sites being covered by TEs is also germane. This fraction is indeed less than you would expect by chance alone given that the genome consists of ~50% TE-derived sites. Clearly, not all TE-derived sites in the human are functionally relevant in terms of expression and many accumulate simply by virtue of the selfish replicative properties of the elements, i.e. without regard to any adaptive benefit they provide to the host genome. In fact most TE insertions into regulatory regions are probably deleterious, and this is consistent with previous results that have shown exclusion of TE sequences from proximal promoter regions. Nevertheless, the fact that a substantial fraction of human gene regulatory sites is derived from TE-sequences underscores the potential for such elements to be co-opted, from time-to-time, to serve some role for the host genome in which they reside*.

*In the Gene function section, we have clarified that genes with TE-derived HS sites were clustered by k-means analysis of their tissue-specific expression patterns. Clusters with pronounced CD4+ T cell expression levels (e.g*. Figure 5)*were then selected for functional analysis with GO. In addition, as per Dr. Gelfand's suggestion, we explicitly point out that we describe an example, not an exhaustive list, in the last paragraph of this section (i.e. the data in *Table 2).

## Competing interests

The author(s) declare that they have no competing interests.

## Authors' contributions

LMR and IKJ performed all analyses. IKJ drafted the manuscript. All authors read and approved the final manuscript.
